# Sustained low abscisic acid levels increase seedling vigor under cold stress in rice (*Oryza sativa* L.)

**DOI:** 10.1038/srep13819

**Published:** 2015-09-09

**Authors:** Ryosuke Mega, Ayano Meguro-Maoka, Akira Endo, Etsuo Shimosaka, Seiji Murayama, Eiji Nambara, Mitsunori Seo, Yuri Kanno, Suzanne R. Abrams, Yutaka Sato

**Affiliations:** 1 Crop Breeding Research Division, National Agriculture and Food Research Organization (NARO), Hokkaido Agricultural Research Center, Hitsujigaoka 1, Toyohira-ku, Sapporo 062-8555, Japan; 2 Centre for the Analysis of Genome Evolution and Function, University of Toronto, Toronto, Ontario M5S 3B2, Canada; 3 Dormancy and Adaptation Research Unit, RIKEN Center for Sustainable Resource Science, Yokohama, Kanagawa 230-0045, Japan; 4 Department of Chemistry, University of Saskatchewan, 110 Science Place, Saskatoon, Saskatchewan S7N 5C7, Canada

## Abstract

Stress-induced abscisic acid (ABA) is mainly catabolized by ABA 8′-hydroxylase (ABA8ox), which also strictly regulates endogenous ABA levels. Although three members of the ABA8ox gene family are conserved in rice, it is not clear which stressors induce expression of these genes. Here, we found that *OsABA8ox1* was induced by cold stress within 24 h and that *OsABA8ox2* and *OsABA8ox3* were not. In contrast, *OsABA8ox2* and *OsABA8ox3* were ABA-inducible, but *OsABA8ox1* was not. *OsABA8ox1*, *OsABA8ox2*, and *OsABA8ox3* restored germination of a *cyp707a1*/*a2*/*a3* triple mutant of *Arabidopsis* to rates comparable to those of the wild type, indicating that *OsABA8ox1*, *OsABA8ox2*, and *OsABA8ox3* function as ABA-catabolic genes *in vivo*. Transgenic rice lines overexpressing *OsABA8ox1* showed decreased levels of ABA and increased seedling vigor at 15 °C. These results indicate that sustained low levels of ABA lead to increased seedling vigor during cold stress. On the other hand, excessively low endogenous ABA levels caused reduced drought and cold tolerance, although some of the transgenic rice lines expressing *OsABA8ox1* at moderate levels did not show these harmful effects. Adequate regulation of endogenous ABA levels is thought to be crucial for maintaining seedling vigor under cold stress and for cold and drought tolerance in rice.

Rice is a globally important, staple crop. Environmental stressors such as low temperature, drought, heat, and high salinity affect rice growth and grain yield. In particular, cold stress causes early seedling growth retardation in rice cultivated in temperate areas^1^. Seedling vigor is mainly defined by rapid growth of shoots and roots in the early vegetative stage^2^,^3^. Generally, inhibition of seedling growth by exposure to low temperatures causes retardation of flowering, heading, and ripening. In addition, rice grain yields are reduced as a result of these delays, because plants are forced to mature under cold temperatures in late fall. Despite these adverse effects on crop yield, little is known about the physiological effects of low temperatures on seedling growth in rice.

Production of abscisic acid (ABA), a plant stress hormone, is increased by cold and drought and acts to help plants withstand these conditions^4^,^5^,^6^,^7^. Changes in ABA levels in response to drought stress control stomatal action: increased levels of ABA trigger stomatal closure, which reduces transpiration under drought conditions, and rehydration decreases ABA levels, leading to stomatal opening^8^. Under conditions of high humidity, an ABA-catabolic gene is specifically expressed in guard cells to promote transpiration in *Arabidopsis thaliana*^9^. Thus, ABA levels in the stomata regulate stomatal action in response to drought. However, the function of ABA with regard to cold stress remains unclear.

There are fewer data for ABA in rice than in *Arabidopsis*. An ABA-deficient mutant of *Arabidopsis* that exhibited severe growth retardation was found^10^, but no such mutant has been obtained yet in rice. Alternatively, several carotenoid (an ABA biosynthetic precursor molecule) deficient rice mutants showed strong cold tolerance, larger stomatal aperture, and earlier wilting than wild-type rice, at both the seedling and panicle development stages^11^. Furthermore, transgenic rice lines with anther-specific overexpression of a wheat ABA catabolic gene demonstrated reduced sterility caused by cold stress during the pollen developmental stage, when compared with each null-segregant line^12^. These findings suggest that high ABA levels negatively affect cold stress tolerance, in contrast to their positive effects in drought conditions. Therefore, rice seedlings might grow more vigorously if ABA levels are sustained at low levels under cold stress, although the physiological mechanisms promoting seedling vigor under cold stress could be different from those providing cold tolerance at the booting stage.

ABA 8′-hydroxylase (ABA8ox) oxidizes ABA to 8′-hydroxy-ABA, which is later spontaneously isomerized to phaseic acid (PA). PA is further reduced to dihydrophaseic acid (DPA) by an unknown reductase^13^,^14^,^15^. In *Arabidopsis*, the genes encoding *ABA8ox* (*CYP707A1*, *CYP707A2*, *CYP707A3*, and *CYP707A4*) have been identified and are known to possess catabolic enzyme activity^16^,^17^. In the case of ABA catabolism, the hydroxylation pathway that converts ABA to PA is predominant in higher plants^18^. Another ABA-inactivation pathway exists, in which ABA is conjugated with glucose to produce ABA glucosyl ester (ABA-GE)^19^. ABA-GE can then be catabolized back to ABA by β-glucosidase^20^. Although three ABA8ox genes are conserved in the rice genome (*OsABA8ox1*, *OsABA8ox2*, and *OsABA8ox3*), only *OsABA8ox1* has been biochemically demonstrated to encode ABA 8′-hydroxylase^21^.

Here, we analyzed the expression of *OsABA8ox1*, *OsABA8ox2*, and *OsABA8ox3* under several stress conditions, and we tested for compensation for these genes with a *cyp707a1*/*cyp707a2*/*cyp707a3* triple mutant of *Arabidopsis*^22^. To analyze the relationships between ABA levels and seedling vigor under cold stress, we produced transgenic rice plants overexpressing *OsABA8ox1*. These transgenic plants had reduced ABA levels and improved seedling vigor under cold stress.

## Results

### Expression of *OsABA8ox* genes under stress conditions in rice

**Figure 1:**
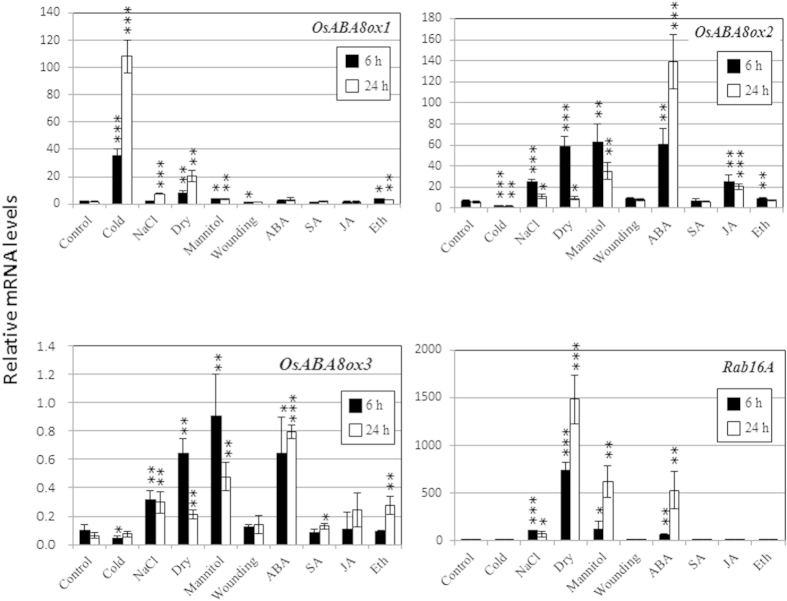
qRT-PCR analysis of *OsABA8ox1*, *OsABA8ox2*, *OsABA8ox3*, and *Rab16A* under stress conditions and hormone treatments. One-week-old seedlings of rice were used for these experiments. For drought treatment (Dry), seedlings were dehydrated on a paper towel. For hormone or osmotic stress treatment, plants were transferred to buffer containing 100 μM abscisic acid (ABA), 100 μM salicylic acid (SA), 100 μM jasmonic acid (JA), 100 μM ethephon (Eth), 250 mM NaCl, or 500 mM mannitol. For cold treatment, plants were transferred to and kept at 4 °C for 6 h or 24 h. For wounding, shoots were punctured by a needle and grown hydroponically in the buffer for 6 or 24 h. Gene expression values are means (±SD) of three biological replicates. Significant differences relative to controls were evaluated using Student’s *t*-test. *, **, and *** represent *P* < 0.05, *P* < 0.01, and *P* < 0.001, respectively.

In rice, three *OsABA8ox* genes that encode ABA 8′-hydroxylases have been revealed by phylogenetic analysis^23^. *Arabidopsis* contains four genes for ABA 8′-hydroxylases (*CYP707A1*, *CYP707A2*, *CYP707A3*, and *CYP707A4*) that are induced by various stressors^9^,^24^,^25^. To examine stress induction of *OsABA8ox* genes in relation to diverse abiotic conditions, we analyzed expression of *OsABA8ox1*, *OsABA8ox2*, and *OsABA8ox3* under cold (4 °C), dehydration, high salt (250 mM NaCl), osmotic stress (500 mM mannitol), and wounding, and following exposure to 100 μM ABA, 100 μM salicylic acid, 100 μM jasmonic acid, and 100 μM ethephon. qRT-PCR showed that expression of *OsABA8ox1*, but not of *OsABA8ox2* or *OsABA8ox3*, was drastically increased by cold stress (Fig. 1). However, *OsABA8ox2* and *OsABA8ox3* were substantially induced by ABA, while *OsABA8ox1* was not. All three genes were induced by high salinity, drought, and osmotic stress, but induction of *OsABA8ox1* was much less than that of *OsABA8ox2* or *OsABA8ox3*. Expression of the ABA-responsive gene *Rab16A* was similar to that of *OsABA8ox2* and *OsABA8ox3* (Fig. 1).

To elucidate the cold inducibility of *OsABA8ox1*, we investigated the 1.1–1.5 kb promoter regions of each *OsABA8ox* gene, and it was found to harbor the low temperature responsive elements LTRE1HVLT49 and LTREATLTI78, according to the PLACE database (Supplementary Fig. S1). In contrast, these elements were not found in the promoter regions of *OsABA8ox2* or *OsABA8ox3* (Supplementary Fig. S1). This suggests that the low temperature responsive elements provide the attribute of cold inducibility to the *OsABA8ox1* gene.

### Rescue of the *cyp707a1*/*cyp707a2*/*cyp707a3* triple mutant phenotype of *Arabidopsis* by rice *OsABA8ox* genes

**Figure 2:**
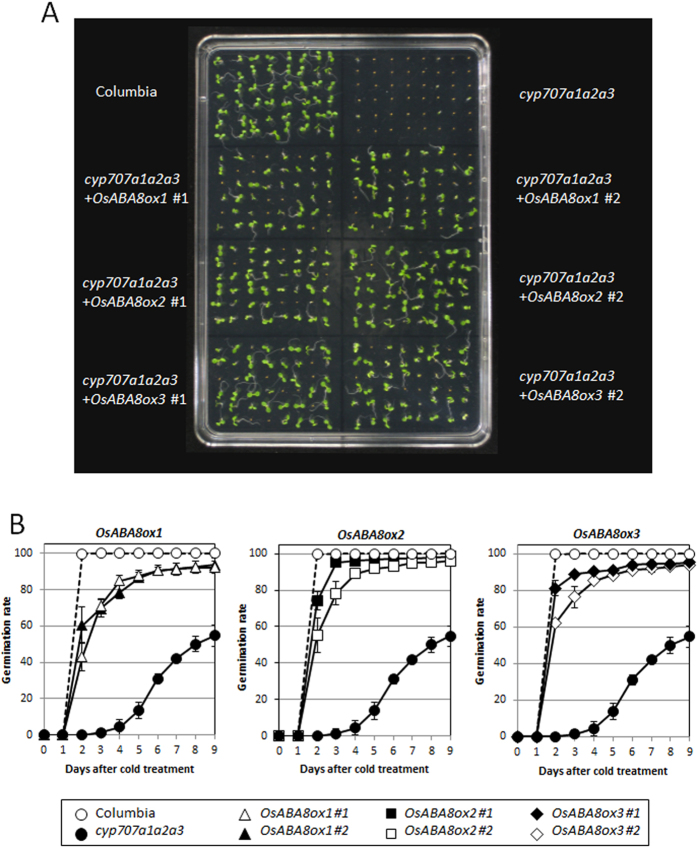
Complementation by *OsABA8ox1*, *OsABA8ox2*, or *OsABA8ox3* in the *cyp707a1a2a3* triple mutant. (**A**) Typical images of germination of wild-type (Columbia), triple mutant, and transgenic plants. (**B**) Germination rates of wild-type, *cyp707a1a2a3*, and transgenic plants after cold treatment. Values are means ± SD of four replicates.

To verify whether rice *OsABA8ox* genes possess ABA 8′-hydroxylase function like the *Arabidopsis cyp707a* genes, we overexpressed *OsABA8ox1*, *OsABA8ox2*, or *OsABA8ox3* in a *cyp707a1*/*cyp707a2*/*cyp707a3* triple mutant (*Cyp707a1a2a3*) of *Arabidopsis*. *Cyp707a1a2a3* is extremely difficult to germinate because of excessive ABA accumulation^22^. We assessed ABA 8′-hydroxylase function by comparing the germination rates of these transgenic lines with those of non-transgenic *cyp707a1a2a3* and wild-type *Arabidopsis*. Germination of *cyp707a1a2a3* was recovered by overexpression of *OsABA8ox1*, *OsABA8ox2*, or *OsABA8ox3* (Fig. 2). These results indicate that rice *OsABA8ox1*, *OsABA8ox2*, and *OsABA8ox3* can function as ABA 8′-hydroxylases *in vivo*.

### *OsABA8oxs* expression and ABA content under various temperature conditions in rice

**Figure 3:**
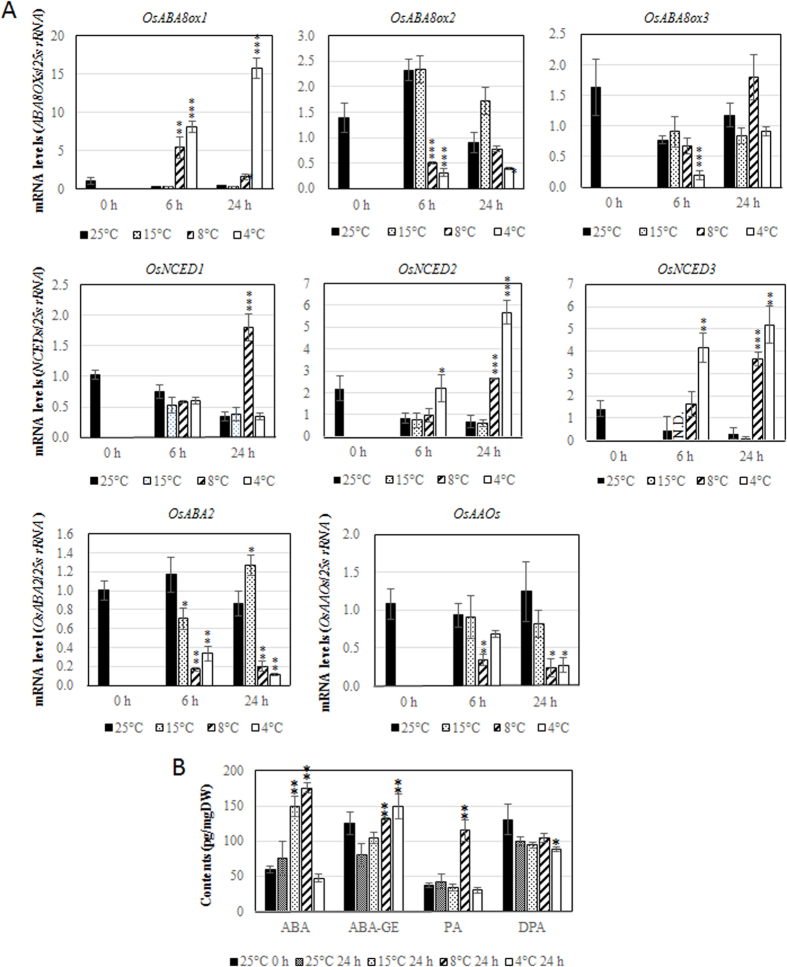
(**A**) Temperature-dependent expression of *OsABA8oxs*, *OsNCEDs*, *OsABA2*, and *OsAAOs*. (**B**) Endogenous ABA, ABA-GE, PA, and DPA content. Three-day-old rice seedlings grown hydroponically were transferred to and kept at 4 °C, 8 °C, 15 °C, and 25 °C for 24 h. Expression of ABA-related genes and endogenous ABA and catabolite contents are means ± SD of three biological replicates. Significant differences between each treatment and the 25 °C treatment were determined by Student’s *t*-tests. *, **, and *** represent *P* < 0.05, *P* < 0.01, and *P* < 0.001, respectively.

To investigate the effects of temperature on expression of *OsABA8oxs*, we cultivated seedlings at various temperatures and then isolated total RNA for qRT-PCR. *OsABA8ox1* expression was strongly induced after cold stress at 4 °C or 8 °C; in contrast, the expression of *OsABA8ox2* and *OsABA8ox3* decreased (Fig. 3A). The 15 °C treatment did not induce expression of *OsABA8ox1* (Fig. 3A). To comprehensively assess ABA metabolism under cold stress, we analyzed mRNA levels of ABA synthesis genes using qRT-PCR; the genes were differentially expressed according to temperature and time (Fig. 3A).

ABA and its catabolites were analyzed by liquid chromatography (LC) tandem mass spectrometry (MS/MS). After 4 °C treatment for 24 h, ABA-GE increased, but ABA, PA, and DPA contents did not change substantially (Fig. 3B) despite significant induction of *OsNCED2*, *OsNCED3*, and *OsABA8ox1* expression by this treatment (Fig. 3A), suggesting that most ABA-biosynthetic and catabolic activities were suppressed at 4 °C at the post-transcriptional levels. Contents of ABA, ABA-GE, and PA increased after treatment at 8 °C (Fig. 3B), consistent with the significant induction of ABA-biosynthetic genes and transient induction of *OsABA8ox1* after 8 °C treatment (Fig. 3A). After 15 °C treatment, ABA content was increased but expression of *OsNCEDs*, thought to be the limiting step of ABA biosynthesis in plants, was not changed. However, expression of *OsABA2* (the downstream gene of *OsNCEDs*) was increased (Fig. 3A). PA and DPA contents did not change after treatment at 15 °C, consistent with the non-induction of *OsABA8ox1* at this temperature (Fig. 3B).

### Growth, *OsABA8ox1* expression, and ABA content under long-term cold stress

Because we found that *OsABA8ox1* was not induced at 15 °C and that ABA content increased at this temperature (Fig. 3B), we measured shoot growth, ABA content, and expression changes of *OsABA8ox1* and other ABA metabolic genes under long-term cold stress to confirm the effect of ABA accumulation.

**Figure 4:**
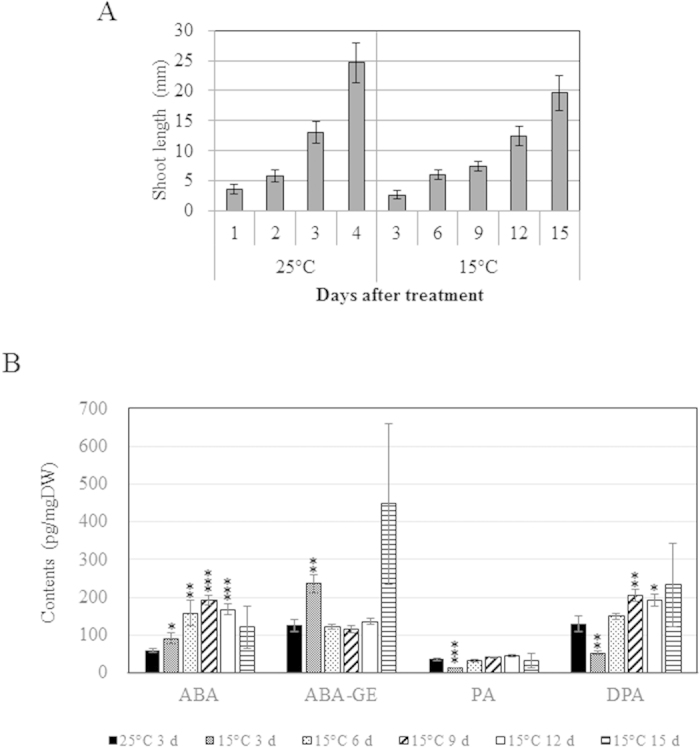
Growth analysis and ABA content under long-term cold stress. Rice seedlings after imbibition were transferred to and grown hydroponically at 15 °C for 15 d. (**A**) Shoot length in seedlings grown at 15 °C or 25 °C after imbibition. Values are means ± SD of nine biological replicates. (**B**) Endogenous ABA, ABA-GE, PA, and DPA contents. Values are means ± SD of three biological replicates. Significant differences between each treatment and the 25 °C treatment were determined by Student’s *t*-tests. *, **, and *** represent *P* < 0.05, *P* < 0.01, and *P* < 0.001, respectively.

Shoot growth of rice seedlings grown at 15 °C was substantially retarded compared to seedlings grown at 25 °C (Fig. 4A). ABA contents increased under cold treatment, reaching a maximum at 9 d (Fig. 4B). ABA-GE contents increased, and PA and DPA contents decreased, after 3 d of cold treatment; and DPA contents generally increased after cold treatment (Fig. 4B).

*OsABA8ox1* expression increased and then declined during cold treatment (Supplementary Fig. S2). Expression of *OsABA8ox2* and *OsABA8ox3* after cold treatment was higher than that of control seedlings grown at 25 °C (Supplementary Fig. S2). Thus, only *OsABA8ox1* tended to be suppressed at 15 °C. Expression of *OsNCED1–3* was suppressed by cold stress, and expression of *OsABA2* and *OsAAOs* was substantially induced after 3 d of cold treatment (Supplementary Fig. S2).

### Effects of overexpression of *OsABA8ox1* on rice seedling vigor under cold conditions

**Figure 5:**
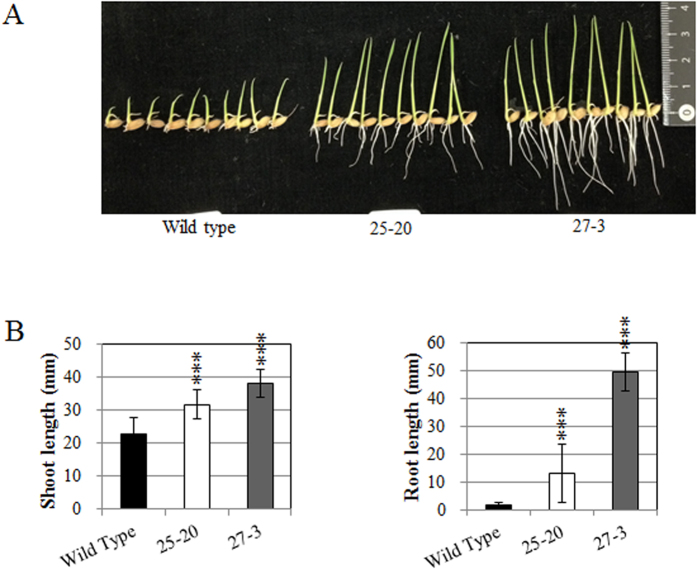
Growth of transgenic rice seedlings overexpressing *OsABA8ox1* and wild-type seedlings (control) at cold temperatures. (**A**) Typical seedlings of Toyohikari (wild type) and transgenic lines overexpressing *OsABA8ox1* at 15 °C. (**B**) Shoot and root length of seedlings grown at 15 °C for 15 days. Values are means ± SD of 27 biological replicates. Significant differences between transgenic and wild-type seedlings were determined by Student’s *t*-tests. *** represent *P* < 0.001.

**Figure 6:**
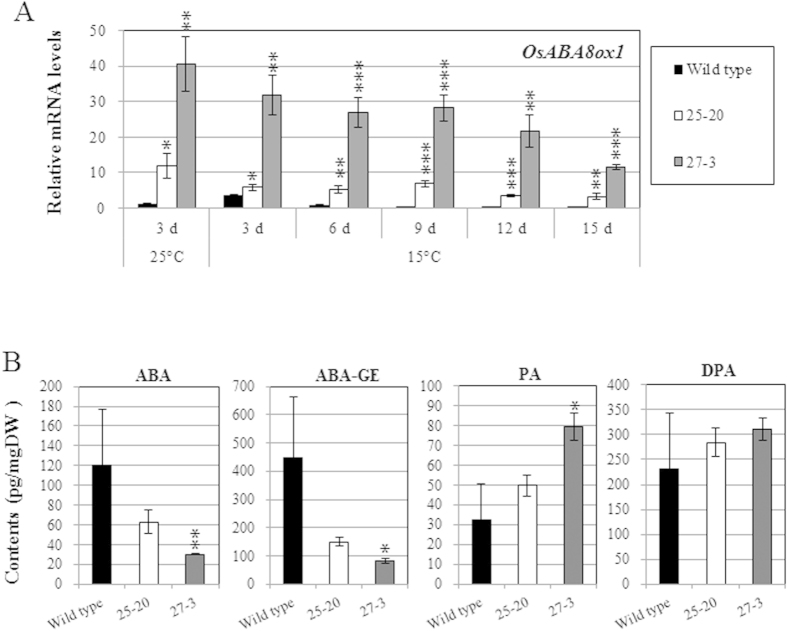
Expression of *OsABA8ox1* and ABA content in wild-type rice seedlings and transgenic rice seedlings overexpressing *OsABA8ox1*. (**A**) qRT-PCR analysis of *OsABA8ox1* in wild-type and transgenic seedlings (25–20: E0082::ABA8ox1 25–20, 27–3: E0082::ABA8ox1 27–3) grown at 25 °C and 15 °C. (**B**) Endogenous ABA, ABA-GE, PA, and DPA contents in wild-type and transgenic seedlings (25–20, 27–3) grown at 25 °C and 15 °C. *OsABA8ox1* gene expression and endogenous Values are means ± SD of three biological replicates. Significant differences between transgenic and wild-type seedlings were determined by Student’s *t*-tests. *, **, and *** represent *P* < 0.05, *P* < 0.01, and *P* < 0.001, respectively.

To analyze the relationships between endogenous ABA levels and seedling vigor under cold conditions, we generated transgenic rice plants overexpressing *OsABA8ox1* and compared the vigor of transgenic lines E0082::ABA8ox1 25–20 (25–20) and E0082::ABA8ox1 27–3 (27–3) with that of wild-type rice (Toyohikari) at 15 °C. Shoots and roots of 25–20 and 27–3 were longer than those of the wild-type seedlings (Fig. 5A,B), and qRT-PCR showed that expression of *OsABA8ox1* was higher in 25–20 and 27–3 (Fig. 6A). ABA and ABA-GE levels were lower in transgenic than in wild-type seedlings, and PA contents were higher in 27–3 than in wild type (Fig. 6B). We also compared seedling vigor and expression of *OsABA8ox1* in two other transgenic lines (E0082::ABA8ox1 11–22 and E0082::ABA8ox1 49–38) with that of wild type and observed results similar to those for 25–20 and 27–3 (Supplementary Fig. S3).

### Effects of overexpression of *OsABA8ox1* on drought and cold stress tolerance

**Figure 7:**
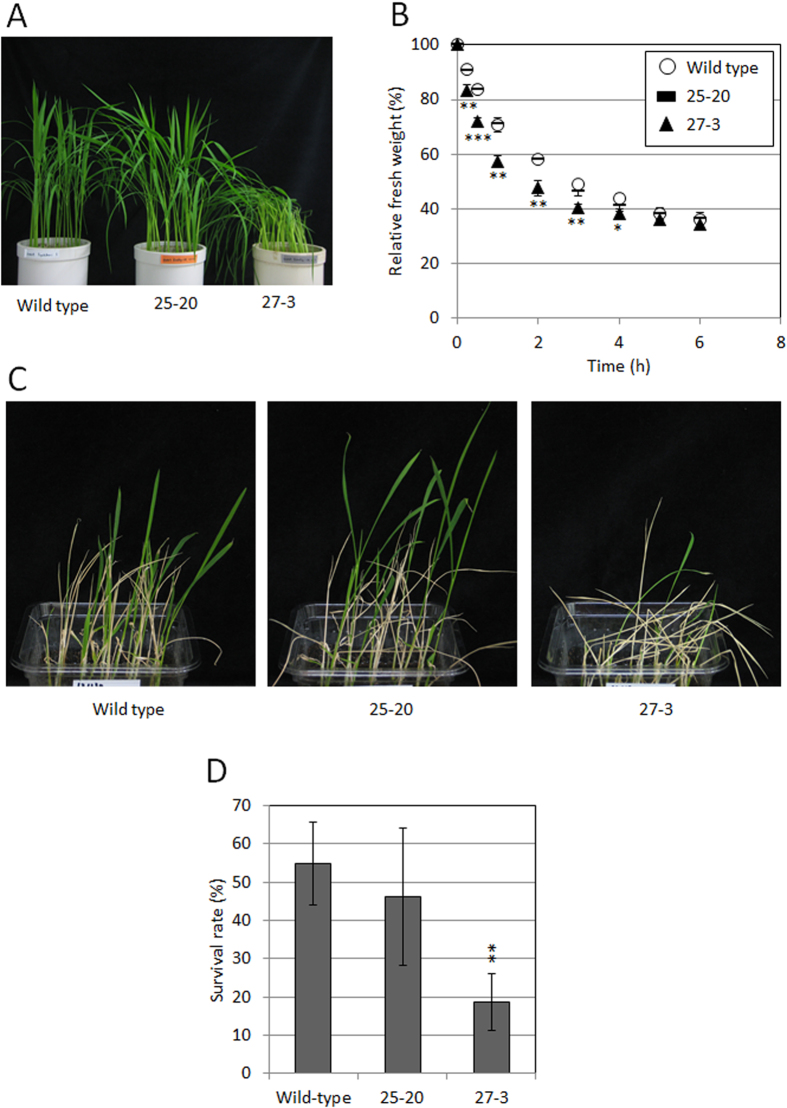
Drought and cold stress tolerance of 25–20 and 27–3. (**A**) Typical plants of Toyohikari (wild type) and transgenic lines overexpressing *OsABA8ox1* were grown in a greenhouse at 25 °C/20 °C. (**B**) Drought stress tolerance of Toyohikari (wild type) and transgenic lines overexpressing *OsABA8ox1*. The weight of harvested leaves was measured at intervals of 15 min, 30 min, or 1 h. Relative fresh weight was calculated to estimate water loss; values are means ± SD of three biological replicates. Significant differences relative to the wild type were determined by Student’s *t*-tests. *, **, and *** represent *P* < 0.05, *P* < 0.01, and *P* < 0.001 respectively. (**C**) Typical images of wild-type, 25–20, and 27–3, 2 weeks after cold treatment (4 °C, 5 d). (**D**) Survival rates of wild-type, 25–20, and 27–3, 2 weeks after cold treatment (4 °C, 5 d). The experiments were repeated four times. Significant differences relative to the wild type were determined by Student’s *t*-tests. **, *P* < 0.01.

Drought and cold stress tolerance of 25–20 and 27–3 were compared with those of wild-type rice (Toyohikari). The relative fresh weight of leaves was found to be substantially lower in 27–3 than in wild-type plants and 25–20, 15 min to 4 h after harvest (Fig. 7A,B). Thus, drought tolerance of 27–3 was shown to be lower than that of wild-type plants or 25–20. The survival rate of seedlings of 27–3 after cold treatment (4 °C, 5 d) was substantially lower than that of seedlings of in wild-type or 25–20 (Fig. 7C,D). Therefore, cold stress tolerance at the seedling stage of 27–3 was lower than that of wild-type plants or 25–20.

### Microarray analysis

**Figure 8:**
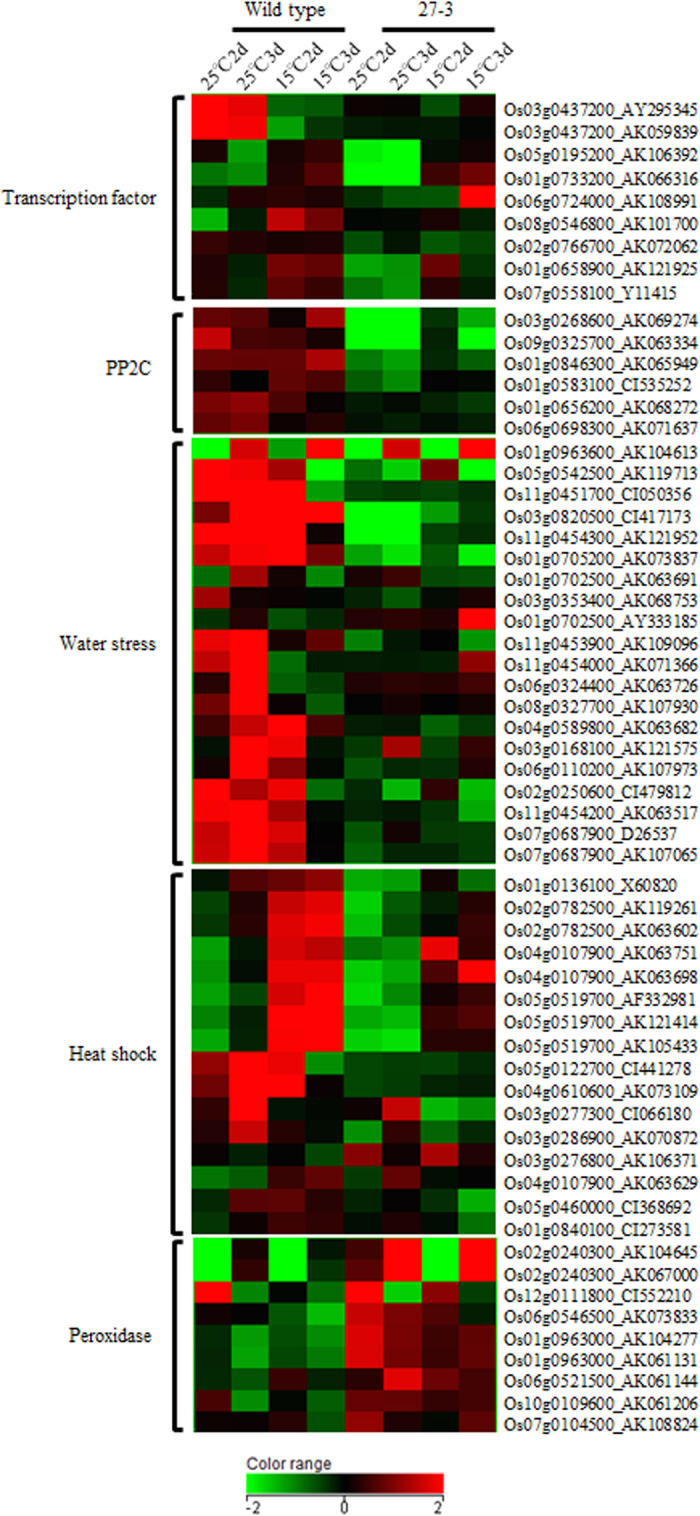
Heatmap of differentially expressed genes in wild-type seedlings and transgenic seedlings overexpressing *OsABA8ox1* (27–3). Genes were selected based on fold changes (>2) between wild-type and transgenic seedlings grown at 15 °C or 25 °C. Then, genes that were reported in the database (RiceXPro, http://ricexpro.dna.affrc.go.jp/) and in the literature as ABA responsive were further selected (Supplementary Table SII). Results are shown in the heatmap. Genes are categorized by function (left). Locus and Accession Nos. are shown on the right.

Genes with differential expression between transgenic rice seedlings (E0082::ABA8ox1 27–3) and wild-type seedlings under cold stress (15 °C) were selected (Fig. 8 and Supplementary Table SII). The genes for transcription factors, including putative genes for ABA-responsive element binding factors, were induced lower in 27–3 by cold stress than that in wild type. Expression of PP2C genes and water-stress related genes, including dehydrin RAB and LEA protein genes, were lower in 27–3 than in wild type at both 25 °C and 15 °C. Heat shock-related genes, including HSP genes, were induced to a lesser extent by cold stress in 27–3 than in wild type. In contrast, peroxidase genes were expressed to a greater extent in 27–3 than in wild type at both 25 °C and 15 °C.

## Discussion

### Stress-dependent expression of *OsABA8ox1*, *OsABA8ox2*, and *OsABA8ox3*

In this study, we showed that expression of *OsABA8ox1* was induced by cold temperatures, while expression of *OsABA8ox2* and *OsABA8ox3* was not. In contrast, expression of *OsABA8ox2* and *OsABA8ox3* was induced by ABA, but *OsABA8ox1* was not. In addition, expression of *OsABA8ox2* and *OsABA8ox3* was induced at much higher levels than that of *OsABA8ox1* by drought, high salinity, and osmotic stress (Fig. 1). These results suggest that *OsABA8ox1* is a cold-inducible gene, whereas *OsABA8ox2* and *OsABA8ox3* are ABA-inducible genes with expression similarly to that of the ABA-responsive gene *Rab16A*. The differential inducibility among the *OsABA8ox* homologs may be unique to rice, because the *Arabidopsis CYP707A1*, *CYP707A2*, *CYP707A3*, and *CYP707A4* genes are all induced by ABA, drought, high salinity, and osmotic stress^17^. Expression of these *Arabidopsis CYP707A* genes also increases in cauline leaves at 0 °C^26^. Thus, all ABA8ox homologs in *Arabidopsis* are induced by the same stressors. However, we demonstrated that the inducibility of *OsABA8ox1* was different from that of *OsABA8ox2* or *OsABA8ox3* in rice, suggesting that the evolutionary origin of *OsABA8ox1* may be different from that of *OsABA8ox2* and *OsABA8ox3*. Phylogenetic analyses classify rice *OsABA8ox2* and *OsABA8ox3* into extremely close clades and place *OsABA8ox1* in a different group^23^. In addition, *OsABA8ox1* consists of 4 exons, whereas *OsABA8ox2* and *OsABA8ox3* consist of 9 and 8 exons, respectively. These findings suggest that *OsABA8ox1* evolved in a manner distinct from *OsABA8ox2* and *OsABA8ox3* in rice.

As noted in Fig. 1, *OsABA8ox1* is thought to play a pivotal role in responses to cold stress in rice. However, *OsABA8ox*2 and *OsABA8ox*3 function under different stress conditions (e.g., drought, ABA, high salinity, and osmotic stress). Since *OsABA8ox3* is induced within 3 to 6 h of seed imbibition^27^, *OsABA8ox3* might help promote seed germination by catabolizing ABA after imbibition.

### *In vivo* ABA catabolic ability of *OsABA8ox1*, *OsABA8ox2*, and *OsABA8ox3*

Overexpression of *OsABA8ox1*, *OsABA8ox2*, or *OsABA8ox3* resulted in the recovery of defective germination in the *Arabidopsis cyp707a1a2a3* triple mutant, in which excess accumulation of ABA occurs in the seeds (Fig. 2). This indicates that the gene products of rice OsABA8ox1, OsABA8ox2, and OsABA8ox3 can catabolize ABA *in vivo*. However, the germination rates of all transgenic lines failed to reach the same level as that of wild-type plants, indicating that the three *Arabidopsis cyp707a* genes might function cooperatively. Although ABA levels of *cyp707a1* or *cyp707a2* single mutants of *Arabidopsis* are similar to those of wild-type plants, ABA levels of the *cyp707a1*/*cyp707a2* double mutant increase 5- to 10-fold relative to those of the *cyp707a1* or *cyp707a2* single mutants^24^. Therefore, even though seed germination of *cyp707a1a2a3* was rescued by overexpression of each single *OsABA8ox* gene, multiple genes might be required to completely recover the same germination rate as the wild-type plant. Alternatively, the rice *OsABA8ox* genes may not function the same as *Arabidopsis ABA8ox* genes *in vivo*.

### Effects of lowered endogenous ABA levels on seedling vigor under cold stress

Comparisons of seedling vigor at 15 °C between wild-type seedlings and 25–20 and 27–3 transgenic seedlings revealed that overexpression of *OsABA8ox1* increased seedling vigor under cold conditions (Fig. 5A,B). The expression level of *OsABA8ox1* in 27–3 was even higher than that in 25–20 (Fig. 6A), and, as predicted, the endogenous ABA level in 27–3 was lower than that in 25–20 (Fig. 6B), and seedling vigor at 15 °C was superior in 27–3. Hence, overexpression of *OsABA8ox1* resulted in lower levels of endogenous ABA, which in turn led to increased seedling vigor under cold conditions. These results suggest that growth of rice seedlings is inhibited by increased ABA levels during cold conditions, and that this inhibition can be blocked by lowering ABA levels. Research to date indicates that inhibition of carotenoid biosynthesis contributed to cold stress tolerance in the vegetative stage in rice^11^, and that overexpression of anther-specific wheat *ABA8ox* reduced seed sterility caused by exposure to cold stress^12^. Recently, we demonstrated that severe ABA inhibition of rice seedling growth was completely blocked by salicylic acid, which showed strong antagonistic effects towards ABA^28^. We found that expression of many stress-related genes was lowered or was not induced by cold stress, while peroxidase genes had higher expression in transgenic rice seedlings overexpressing *OsABA8ox1* than in wild-type seedlings (Fig. 8 and Supplementary Table SII). Plant peroxidases can induce cell wall loosening and growth by elongation and cross-linking of cell wall components^29^. Seedling vigor might have been improved by release of ABA suppression against peroxidase genes in transgenic seedlings overexpressing *OsABA*80*x*1. Thus, reducing ABA levels or its effects might be a key mechanism for conferring tolerance to cold conditions to rice.

Although we succeeded in increasing seedling vigor under cold conditions, transgenic rice plants overexpressing *OsABA8ox1* showed a few disadvantageous characteristics. First, drought tolerance of 27–3 was substantially lower than that of wild-type and 25–20 (Fig. 7A,B), which we suggest was caused by the extremely low endogenous ABA levels. One report demonstrated that tobacco plants overexpressing three *ABA*8*ox* genes from the common bean (*Phaseolus vulgaris* L.) had wilty phenotypes and broadly opened stomata compared to non-transgenic tobacco, and that these phenotypes were the result of reduced endogenous ABA levels^30^. ABA helps stomata to close and thus to reduce transpiration under drought conditions, and ABA degradation promotes stomatal opening^8^. Here, 27–3 probably wilted because constitutive *OsABA8ox1* overexpression decreased endogenous ABA levels excessively, forcing stomata to remain open. Several ABA-deficient lines in *Arabidopsis* also show an extreme wilty phenotype under normal conditions^10^. In contrast, 25–20 showed growth that was similar to wild-type plants although endogenous ABA was reduced. These differences between 25–20 and 27–3 are thought to be caused by the differences in endogenous ABA levels. The ABA content in 27–3 was 30 pg/mg dry weight (DW), which was substantially lower than that of 25–20 (60 pg/mg DW) (Fig. 6B). Thus, the wilty phenotype of 27–3 might be caused by the excessively reduced ABA levels. Measuring the width of stomatal aperture might help to determine this threshold. Secondly, the survival rate after cold treatment (4 °C) in 27–3 was inferior to that of wild-type or 25–20 (Fig. 7C,D). This result was inconsistent with the increased cold tolerance of the carotenoid-deficient mutant^11^ and might be a result of excessive degradation of endogenous ABA. We found that the expression of many stress-related genes was lowered or not induced by cold stress in 27–3 compared to wild type (Fig. 8 and Supplementary Table SII), which could affect the survival rate of 27–3 after cold treatment (4 °C). In contrast, 25–20 showed almost the same survival rate as the wild-type strain (Fig. 7C,D). Therefore, excessive ABA degradation might actually reduce cold tolerance. Third, the seed filling rate of 27–3 was lower than that of wild-type or 25–20 under normal conditions (Supplementary Fig. S4), and seed filling rate was similar in 25–20 and wild type. This suggests that mild *OsABA8ox1* overexpression has little effect on rice seed ripening, whereas excessive *OsABA8ox1* expression results in increased seed sterility. Taken together, our findings suggest that adequate regulation of endogenous ABA levels in rice can lead to improved seedling vigor under cold temperatures without lowering cold and drought tolerance.

## Conclusion

The findings presented here provide important knowledge toward understanding the relationships between endogenous ABA levels and seedling vigor under cold conditions in rice. By using transgenic rice plants overexpressing *OsABA8ox1*, we demonstrated that sustained low levels of endogenous ABA led to increased seedling vigor under cold conditions. On the other hand, excessively low endogenous ABA levels reduced drought and cold tolerance. We propose that adequate regulation of endogenous ABA levels in rice is crucial both for seedling vigor under cold conditions and for cold and drought tolerance.

## Methods

### Plant materials, growth conditions, and treatments

Seeds of rice (Toyohikari) were washed with sterilized water and soaked in water for 2 d at 28 °C in the dark. After soaking, germinated seeds were grown under hydroponic conditions as follows. Nine seeds of Toyohikari were placed on a plastic grid (approximately 35 × 35 mm), which was floated in a plastic cup holding 100 mL distilled water. Seeds were grown hydroponically in water containing 2.5 mM MES-KOH (pH 5.8) in a growth chamber (12 h light: 12 h dark; 25 °C) for 1 week, and 1-week-old seedlings were used for the subsequent experiments. For drought treatment, seedlings were dehydrated on a paper towel. For hormone and osmotic stress treatment, seedlings were transferred to buffer containing either 100 μM ABA, 100 μM salicylic acid, 100 μM jasmonic acid, 100 μM ethephon, 250 mM NaCl, or 500 mM mannitol. For cold treatment, seedlings were transferred to and kept at 4 °C for 6 or 24 h. For wounding, shoots were punctured by a needle. Wounded seedlings were grown hydroponically in the buffer for 6 or 24 h. To investigate the effects of temperature, germinated seeds were hydroponically grown for 3 d as described above and then were transferred to and kept at 4 °C, 8 °C, 15 °C, and 25 °C for 6 or 24 h.

### qRT-PCR

Shoots were harvested from stress-treated seedlings for qRT-PCR. The shoots were frozen with liquid nitrogen and applied to a Multi-beads Shocker (Yasui-Kikai, Osaka) cell disruptor at 1500 rpm for 10 s. Total RNA was isolated using an RNeasy plant mini kit (Qiagen, Venlo), according to the manufacturer’s manual, then treated with DNase I (Takara, Tokyo) to prevent contamination by genomic DNA. cDNA was then synthesized from 500 ng cleaned total RNA using PrimeScript RT Master Mix (Takara). qRT-PCR was performed using TaqMan with the Universal Probe Library (Roche, Basel, Switzerland). Primer sets used in qRT-PCR are provided in Supplementary Table SI. For normalization of data, 25S rRNA was used as an internal standard^31^.

### ABA measurements

ABA and its catabolites were analyzed by liquid chromatography (LC) tandem mass spectrometry (MS/MS). Deuterium-labeled d_6_-ABA purchased from Icon Services (Summit) and deuterium-labeled d_3_-PA, d_3_-DPA, d_5_-ABA-GE, d_3_-neoPA, and d_4_-7′OH-ABA^32^ were used as internal standards. The tissue was frozen in liquid nitrogen and stored at −80 °C. Lyophilized plant materials were ground in 2 ml of 80% acetonitrile containing 1% acetic acid, extraction and purification of ABA and its catabolites were performed as described previously^33^. The hormone and metabolites were quantified by LC (Agilent 1200 UHPLC; Agilent Technologies) and a triple quadrupole mass spectrometer (Agilent 6410; Agilent Technologies). The LC conditions were the same as described previously^33^. The retention times of the compounds were 11.4 min (DPA and d_3_-DPA), 18.3 min (PA and d_3_-PA), 13.6 min (ABA-GE and d_5_-ABA-GE), 21.3 min (7′OH-ABA and d_4_-7′OH-ABA), 23.8 min (neoPA and d_3_-neoPA), 27.1 min (d_6_-ABA and ABA). MS/MS conditions were as follows: collision energy (eV) = 8.0 (ABA and d_6_-ABA), 10.0 (PA and d_3_-PA, neoPA, and d_3_-neoPA, ABA-GE and d_5_-ABA-GE), 12.0 (7′OH-ABA and d_4_-7′OH-ABA), 16.0 (DPA, d_3_-DPA) and fragmentor (V) = 140 (ABA and d_6_-ABA, DPA and d_3_-DPA), 130 (PA and d_3_-PA, neoPA, and d_3_-neoPA), 160 (ABA-GE and d_5_-ABA-GE), 110 (7′OH-ABA and d_4_-7′OH-ABA). MS/MS transitions (m/z) were: 263/153 (ABA), 269/159 (d_6_-ABA), 279/139 (PA), 282/142 (d_3_-PA), 281/171 (DPA), 284/174 (d_3_-DPA), 425/263 (ABA-GE), 430/268 (d_5_-ABA-GE), 279/217 (7′OH-ABA), 283/221 (d_4_-7′OH-ABA), 279/205 (neoPA), and 282/208 (d_3_-neoPA).

### Introduction of *OsABA8ox1, OsABA8ox2*, or *OsABA8ox3* into *cyp707a1/cyp707a2/cyp707a3* triple mutant of Arabidopsis

*OsABA8ox1*, *OsABA8ox2*, or *OsABA8ox3* cDNA fragments were amplified by PCR from synthesized total cDNA with the following primers: *OsABA8ox1*, ABA8ox1_fw and ABA8ox1_rv; *OsABA8ox2*, ABA8ox2_fw and ABA8ox2_rv; *OsABA8ox3*, ABA8ox3_fw and ABA8ox3_rv (Supplementary Table SI). These fragments were cloned into pENTR/D-TOPO vectors (Life Technologies, Rockville). After nucleotide sequences were checked, the cloned cDNAs were transferred to the binary vector pEarleyGate101 via the Gateway LR Clonase Enzyme mix (Life Technologies)^34^. The resulting plasmids were introduced into *Agrobacterium* strain GV3101, which was used to transform the *cyp707a1a2a3* triple mutant^22^ by the floral-dipping method^35^. Transgenic plants were obtained by selection with Basta. T_2_-independent transgenic lines were analyzed.

### Germination analysis of *Arabidopsis* plants

The *in vivo* function of rice *OsABA8ox* genes was estimated by quantifying the germination rate of transgenic *Arabidopsis* lines. Two hundred seeds were sown on 0.8% agar plates containing one-half Murashige and Skoog salts and 2.5 mM MES-KOH (pH 5.8). After 2 d of cold treatment at 4 °C, the plates were placed at 22 °C under continuous light for 9 d. Germination was scored daily for radicle emergence. The experiments were repeated four times.

### Production of transgenic rice plants overexpressing *OsABA8ox1*

The 1416-bp *OsABA8ox1* cDNA fragment was amplified from synthesized total cDNA with the primers ABA8ox1_fw2 and ABA8ox1_rv2 (Supplementary Table SI). The amplified cDNA fragment was cloned into a pSTARA-R4 vector (Funakoshi, Tokyo). The fragment was inserted downstream of the E0082 promoter^36^ that drives expression in green tissues. Transformation of wild-type rice (Toyohikari) and selection of transgenic rice were performed as described previously^37^.

### Growth analysis of rice seedlings under long-term cold stress

Seeds of rice (Toyohikari) were washed with sterilized water and soaked in water for 2 d at 28 °C in the dark. After soaking, nine seeds each of transgenic rice plants overexpressing *OsABA8ox1* and of wild-type plants were placed on a plastic grid (approximately 35 × 35 mm), which was floated in a plastic cup holding 100 mL distilled water. Plants were grown in the growth chamber (12 h light: 12 h dark; 15 °C) for 15 d.

### Evaluation of drought and cold stress tolerance

For drought stress treatment, plants were grown in soil in a greenhouse (14 h light, 25 °C; 10 h dark, 20 °C) for 1 month, and then the leaves were harvested. The weight of harvested leaves was measured at intervals of 15 min, 30 min, or 1 h. Relative fresh weight was calculated to estimate water loss. For cold stress treatment, 20 plants each of the wild type and transgenic plants were pre-grown in soil in a greenhouse (14 h light, 25 °C; 10 h dark, 20 °C) for 10 d and then transferred to a growth chamber (12 h light, 4 °C; 12 h dark, 4 °C). After the stress treatment for 5 d, plants were returned to the greenhouse and grown for an additional 2 weeks. The numbers of plants that survived and continued to grow were counted. The experiments were repeated four times.

### Microarray analysis

Shoot samples were collected from seedlings grown at 15 °C or 25 °C. Total RNA was extracted as described above (qRT-PCR). The integrity of each RNA sample was examined using lab-on-a-chip technology with the RNA 6000 Nano LabChip kit and a 2100 Bioanalyzer (Agilent Technologies, Santa Clara). Microarrays were performed using a Low-Input Quick Amp Labeling Kit (Agilent Technologies) and a rice 4 × 44 K custom oligo DNA microarray (Agilent Technologies) according to the manufacturer’s instructions. Hybridization microarray slides were scanned with a Microarray Scanner and the resulting images were analyzed using Feature Extraction software (Agilent Technologies), applying standard normalization procedures. The analyses were performed using three biological replicates. Heat maps were generated using GeneSpring 12.5 software.

### Statistics

For all statistical analyses, group data were averaged and standard deviations were calculated. Student’s *t*-tests were used to compare the appropriate experimental and control group means; *P* values <0.05 were considered statistically significant.

## Additional Information

**Accession numbers:** *OsABA8ox1*:Os02g0703600; *OsABA8ox2*:Os08g0472800; *OsABA8ox3*:CI523426; *Rab16A*:Os11g0454300; *25S rRNA*:AK119809; *OsNCED1*:CI049010; *OsNCED2*:Os12g0617400; *OsNCED3*:Os07g0154100; *OsABA2*:Os03g0810800; *OsAAOs*:Os03g0790900/Os10g0138100.

**How to cite this article**: Mega, R. *et al*. Sustained low abscisic acid levels increase seedling vigor under cold stress in rice (*Oryza sativa* L.). *Sci. Rep*. **5**, 13819; doi: 10.1038/srep13819 (2015).

## Supplementary Material

Supplementary Data
