# Supplementary material for: Sustained low abscisic acid levels increase seedling vigor under cold stress in rice (Oryza sativa L.)
Source: Sci Rep. 2015 Sep 9;5:13819. doi: 10.1038/srep13819 (PMC4563555; doi:10.1038/srep13819)
Supplement: Supplementary Data [file srep13819-s1.pdf]

**Sustained low abscisic acid levels increase seedling vigor under cold stress in rice (*Oryza sativa* L.)**

**Ryosuke Mega[a,1], Ayano Meguro-Maoka[a,2], Akira Endo[a,3], Etsuo Shimosaka[a], Seiji Murayama[a,4], Eiji Nambara[b], Mitsunori Seo[c], Yuri Kanno[c] Suzanne R. Abrams[d] and Yutaka Sato[a*]**

[a]Crop Breeding Research Division, National Agriculture and Food Research Organization (NARO), Hokkaido Agricultural Research Center, Hitsujigaoka 1, Toyohira-ku, Sapporo, 062-8555, Japan

[b]Centre for the Analysis of Genome Evolution and Function, University of Toronto, Toronto, Ontario, Canada M5S 3B2

[c]Dormancy and Adaptation Research Unit, RIKEN Center for Sustainable Resource Science, Yokohama, Kanagawa, 230-0045, Japan

[d]Department of Chemistry, University of Saskatchewan, 110 Science Place, Saskatoon, Saskatchewan S7N 5C7, Canada

## Supplementary Data

**Supplementary Table SI.** List of primers used in this study.

**Supplementary Table SII.** Genes with differential expression between transgenic rice (E0082::ABA8ox1 27-3) and wild-type seedlings under cold stress (15°C).

**Supplementary Figure S1.** Distribution of *cis*-acting elements in the promoter region of *OsABA8ox1, OsABA8ox2, and OsABA8ox3*.

**Supplementary Figure S2.** Expression of *OsABA8oxs*, *OsNCEDs*, *OsABA2*, and *OsAAOs* under long-term cold stress.

**Supplementary Figure S3.** Growth and *OsABA8ox1* gene expression of E0082::ABA8ox1 11-22 and E0082::ABA8ox1 49-38.

**Supplementary Figure S4.** Sterility rate of E0082::ABA8ox1 25-20 and E0082::ABA8ox1 27-3.

## Supplementary Table SI. List of primers used in this study

| Experiment | Gene | Locus/Accession No. | Forward primer | Reverse primer | TaqMan probes |
|---|---|---|---|---|---|
| Transgenic Arabidopsis | *OsABA8ox1* | Os02g0703600 | caccatgcccctgcgtgatg | ctcctgctcggtgttcttg | |
| | *OsABA8ox2* | Os08g0472800 | caccatggctttcttgctcttctttgt | agcacctgctgctgtgatg | |
| | *OsABA8ox3* | CI523426 | caccatggcagcctccttcgtc | aaccattgctacgccattgtc | |
| Transgenic rice | *OsABA8ox1* | Os02g0703600 | ggttccgcgtggatccatgggtgct tttcttctgttcg | gtcgacccgggaattctcactcctgc tcggtgttct | |
| QRT-PCR | *OsABA8ox1* | Os02g0703600 | tcaacaccttccaagagatgaa | atctcctcctccccgaag | #56 |
| | *OsABA8ox2* | Os08g0472800 | ggcgagcataatctccttca | ccctttggaatcaggaaacc | #34 |
| | *OsABA8ox3* | CI523426 | catggccctaacccacaa | gggataaggaaccctttgtactc | #159 |
| | *Rab16A* | Os11g0454300 | gagggaggagcacaagacc | attccatcatcctcagacgag | #78 |
| | *25S rRNA* | AK119809 | ctcttttgccccatgcac | agcaatctgtctgctcatcaag | #82 |
| | *OsNCED1* | CI049010 | tcgccatcaccgagaacta | tctcctggagcttgaacacc | #131 |
| | *OsNCED2* | Os12g0617400 | aagaagaaggatgggctgaac | tgatgaacccttcctcgaac | #11 |
| | *OsNCED3* | Os07g0154100 | ccctaccacgtccgtgtc | gcccatggaagtcgtacct | #152 |
| | *OsABA2* | Os03g0810800 | tggagctgcaattagtgagg | ccacagtcgtacacgaggtc | #119 |
| | *OsAAOs* | Os03g0790900/ Os10g0138100 | gctctctccatgccgtatct | ccacacctttcaggtttgct | #157 |

**Supplementary Table SII.** Genes with differential expression between transgenic rice (E0082::ABA8ox1 27-3) and wild-type seedlings under cold stress (15°C)

| ProbeName | Toyohikari | | | | 27-3 | | | | Rice X pro data | | Description | PrimaryAccession | Referance |
|---|---|---|---|---|---|---|---|---|---|---|---|---|---|
| | 25°C2d | 25°C3d | 15°C9d | 15°C15d | 25°C2d | 25°C3d | 15°C9d | 15°C15d | ABA 6h | ABA 12h | | | |
| Transcription factor | | | | | | | | | | | | | |
| RICEGENE13573 | 987.0 | 706.0 | 1595.6 | 1489.0 | 287.4 | 355.8 | 1585.5 | 622.6 | 7.2 | | OSBZ8. [Os01t0658900-01] | Os01g0658900_AK121925 | 1 |
| RICEGENE30459 | 361.2 | 101.7 | 15.3 | 16.2 | 21.2 | 24.2 | 17.7 | 30.0 | 5.9 | | Zinc finger, C2H2-type domain containing protein. [Os03t0437200-01] | Os03g0437200_AY295345 | |
| RICEGENE08765 | 472.9 | 78.3 | 6.6 | 13.8 | 10.7 | 12.4 | 14.6 | 14.6 | 20.2 | | Zinc finger, C2H2-type domain containing protein. [Os03t0437200-01] | Os03g0437200_AK059839 | |
| RICEGENE17191 | 2911.7 | 1108.2 | 3083.6 | 3557.4 | 575.3 | 377.2 | 2601.1 | 2774.9 | 9.9 | 9.2 | Zinc finger, CCCH-type domain containing protein. [Os05t0195200-01] | Os05g0195200_AK106392 | |
| RICEGENE23696 | 964.3 | 661.3 | 1345.5 | 1145.1 | 354.6 | 342.3 | 1090.4 | 665.1 | 7.4 | 6.5 | Similar to Myb-related transcription factor LBM1. [Os07t0558100-00] | Os07g0558100_Y11415 | |
| RICEGENE36837 | 124.3 | 129.3 | 284.0 | 381.4 | 27.0 | 54.1 | 347.6 | 413.0 | 2.3 | 2.1 | Similar to Heat shock transcription factor 29 (Fragment). [Os01t0733200-01] | Os01g0733200_AK066316 | |
| RICEGENE10967 | 138.7 | 362.4 | 1057.8 | 745.0 | 289.4 | 327.8 | 464.4 | 303.7 | 2.7 | 3.7 | Similar to Heat stress transcription factor B-2b. [Os08t0546800-01] | Os08g0546800_AK101700 | |
| RICEGENE04745 | 3739.5 | 3455.3 | 3363.1 | 3607.7 | 1576.9 | 2363.3 | 1941.8 | 1942.8 | 4.7 | 3.2 | Similar to Abscisic acid responsive elements-binding factor (ABA-responsive element binding protein 2) (AREB2). [Os02t0766700-01] | Os02g0766700_AK072062 | |
| RICEGENE27103 | 21.7 | 41.3 | 35.4 | 34.5 | 17.3 | 17.1 | 21.2 | 852.1 | | 2.9 | Similar to Abscisic acid responsive elements-binding factor. [Os06t0724000- | Os06g0724000_AK108991 | |
| PP2C | | | | | | | | | | | | | |
| RICEGENE06434 | 484.4 | 562.8 | 321.0 | 382.6 | 215.5 | 224.5 | 326.0 | 237.3 | 36.7 | 50.4 | Protein phosphatase 2C family protein. [Os06t0698300-01] | Os06g0698300_AK071637 | |
| RICEGENE32168 | 690.3 | 791.7 | 565.9 | 409.5 | 262.9 | 320.9 | 336.0 | 262.1 | 3.9 | 7.0 | Protein phosphatase 2C family protein. [Os01t0656200-01] | Os01g0656200_AK068272 | 2 |
| RICEGENE08607 | 5623.0 | 4478.9 | 7694.8 | 6999.8 | 2230.8 | 1966.7 | 4791.0 | 4121.9 | 6.5 | 6.7 | Similar to Protein phosphatase 2C. [Os01t0583100-01] | Os01g0583100_CI535252 | 2 |
| RICEGENE26038 | 1017.1 | 984.2 | 1017.8 | 1546.3 | 240.7 | 227.6 | 510.7 | 339.4 | 12.2 | 8.5 | Similar to protein phosphatase 2C. [Os01t0846300-02] | Os01g0846300_AK065949 | 2 |
| RICEGENE16735 | 823.3 | 736.4 | 527.6 | 1161.8 | 54.8 | 140.0 | 833.5 | 182.0 | 13.8 | 14.0 | Similar to Protein phosphatase type 2C. [Os03t0268600-01] | Os03g0268600_AK069274 | 2 |
| RICEGENE03743 | 5560.3 | 2837.3 | 2816.3 | 2344.7 | 353.7 | 306.9 | 1776.8 | 277.3 | 75.0 | 60.9 | Similar to Protein phpsphatase 2C (PP2C) (EC 3.1.3.16). [Os09t0325700-01] | Os09g0325700_AK063334 | 2 |
| Water stress | | | | | | | | | | | | | |
| RICEGENE25055 | 2314.5 | 6713.1 | 4723.5 | 7364.1 | 70.5 | 182.0 | 459.7 | 691.6 | 85.5 | 76.7 | Similar to WCOR719. [Os03t0820500-01] | Os03g0820500_CI417173 | |
| RICEGENE15477 | 64.2 | 156.9 | 59.9 | 24.7 | 11.3 | 16.6 | 20.2 | 16.9 | 9.3 | | WSI76 protein induced by water stress. [Os07t0687900-01] | Os07g0687900_AK107065 | |
| RICEGENE37809 | 54.4 | 82.3 | 60.9 | 23.1 | 10.3 | 19.5 | 15.4 | 14.0 | 8.6 | | WSI76 protein induced by water stress. [Os07t0687900-01] | Os07g0687900_D26537 | |
| RICEGENE06777 | 512.3 | 1622.6 | 334.9 | 138.6 | 89.7 | 110.3 | 111.2 | 53.6 | 82.2 | 174.7 | Dehydrin RAB 16B. [Os11t0454200-01] | Os11g0454200_AK063517 | 3 |
| RICEGENE04011 | 59.4 | 522.9 | 9.0 | 17.6 | 11.0 | 12.6 | 14.6 | 68.5 | | | Dehydrin RAB 16C. [Os11t0454000-01] | Os11g0454000_AK071366 | 3 |
| RICEGENE04307 | 95.9 | 1279.3 | 30.4 | 84.8 | 10.9 | 21.6 | 28.4 | 12.3 | | | Dehydrin RAB 16D. [Os11t0453900-01] | Os11g0453900_AK109096 | 3 |
| RICEGENE05739 | 427.3 | 2463.9 | 171.0 | 8.9 | 11.6 | 13.6 | 15.5 | 15.6 | 499.1 | 560.2 | Similar to Dehydrin DHN1 (M3) (RAB-17 protein). [Os11t0451700-00] | Os11g0451700_CI050356 | |
| RICEGENE29579 | 7.1 | 428.7 | 58.4 | 1241.9 | 29.0 | 366.6 | 14.2 | 1871.4 | 2.3 | | ABA/WDS induced protein family protein. [Os01t0963600-01] | Os01g0963600_AK104613 | |
| RICEGENE13810 | 1499.1 | 14149.5 | 3503.6 | 345.4 | 52.2 | 62.1 | 232.6 | 230.2 | 8.0 | 9.5 | Similar to Water-stress inducible protein RAB21. [Os11t0454300-01] | Os11g0454300_AK121952 | |
| RICEGENE02055 | 7.6 | 16.1 | 6.9 | 8.6 | 10.4 | 12.1 | 13.6 | 146.7 | 6.5 | 8.6 | Rep: Dehydrin Rab25 - Oryza sativa subsp. japonica (Rice), complete [TC524743] | Os01g0702500_AY333185 | |
| RICEGENE27617 | 22.9 | 126.6 | 45.0 | 21.5 | 45.6 | 49.8 | 33.7 | 25.2 | 3.9 | 4.0 | Similar to Dehydrin Rab25. [Os01t0702500-01] | Os01g0702500_AK063691 | |
| RICEGENE22964 | 479.0 | 234.0 | 225.2 | 212.4 | 142.0 | 121.3 | 214.2 | 234.8 | 2.3 | | Similar to Early response to dehydration 15-like protein. [Os03t0353400-02] | Os03g0353400_AK068753 | |

| | | | | | | | | | | | | | |
|---|---|---|---|---|---|---|---|---|---|---|---|---|---|
| Heat shock | | | | | | | | | | | | | |
| RICEGENE26600 | 549.8 | 963.5 | 1103.7 | 1446.9 | 204.0 | 246.7 | 758.4 | 332.6 | 11.2 | 9.6 | 16.9 kDa class I heat shock protein 1. [Os01t0136100-01] | Os01g0136100_X60820 | |
| RICEGENE33820 | 770.5 | 1317.9 | 3510.8 | 4105.2 | 306.9 | 648.7 | 1061.7 | 1332.9 | 7.9 | 12.4 | Similar to 17.5 kDa class II heat shock protein. [Os02t0782500-02] | Os02g0782500_AK063602 | |
| RICEGENE07742 | 20821.0 | 30410.9 | 39464.8 | 38549.0 | 21157.5 | 30415.1 | 28387.5 | 15006.9 | 2.2 | 4.0 | Heat shock protein Hsp70 family protein. [Os01t0840100-01] | Os01g0840100_CI273581 | 4 |
| RICEGENE22604 | 11.6 | 9.7 | 13.3 | 8.3 | 46.2 | 11.6 | 30.1 | 13.8 | 2.4 | 5.4 | Heat shock protein Hsp70 family protein. [Os03t0276800-01] | Os03g0276800_AK106371 | |
| RICEGENE37205 | 142.5 | 501.2 | 103.1 | 112.3 | 96.5 | 293.4 | 46.5 | 52.1 | | | Heat shock protein 70. [Os03t0277300-01] | Os03g0277300_CI066180 | 4 |
| RICEGENE20721 | 186.9 | 371.1 | 392.3 | 308.9 | 156.5 | 219.9 | 201.5 | 111.6 | | | Rep: HSP70 - Triticum aestivum (Wheat), complete [TC515901] | Os05g0460000_CI368692 | 4 |
| RICEGENE17769 | 27.9 | 52.4 | 187.8 | 178.7 | 49.7 | 26.1 | 1036.6 | 76.6 | 11.4 | 35.1 | Similar to Heat shock protein 80. [Os04t0107900-03] | Os04g0107900_AK063751 | |
| RICEGENE19320 | 18.1 | 37.5 | 144.5 | 153.9 | 11.1 | 15.8 | 71.6 | 718.8 | 13.8 | 34.3 | Similar to Heat shock protein 82. [Os04t0107900-01] | Os04g0107900_AK063698 | |
| RICEGENE21268 | 7.8 | 8.9 | 20.0 | 26.2 | 9.4 | 42.5 | 16.0 | 14.3 | | 5.2 | Similar to Heat shock protein 82. [Os04t0107900-04] | Os04g0107900_AK063629 | |
| RICEGENE11851 | 127.3 | 213.0 | 976.6 | 1326.4 | 77.3 | 175.4 | 372.1 | 446.4 | 6.2 | 9.7 | Heat shock protein 101. [Os05t0519700-02] | Os05g0519700_AF332981 | |
| RICEGENE33566 | 329.7 | 563.4 | 2977.9 | 4493.9 | 216.5 | 226.1 | 962.2 | 1054.7 | 9.0 | 10.4 | Similar to Heat shock protein 101. [Os05t0519700-03] | Os05g0519700_AK121414 | |
| RICEGENE01989 | 70.4 | 144.0 | 676.2 | 871.7 | 46.5 | 47.9 | 232.4 | 208.1 | | 10.4 | Heat shock protein 101. [Os05t0519700-01] | Os05g0519700_AK105433 | |
| RICEGENE24082 | 45.2 | 138.0 | 64.4 | 8.2 | 9.8 | 11.4 | 13.4 | 13.8 | 69.2 | 95.7 | Similar to Low-temperature induced protein lt101.2. [Os05t0122700-01] | Os05g0122700_CI441278 | |
| RICEGENE34540 | 349.9 | 843.3 | 338.5 | 269.5 | 102.4 | 320.1 | 172.5 | 214.0 | 59.9 | 84.1 | Similar to Low-temperature induced protein lt101.2. [Os03t0286900-01] | Os03g0286900_AK070872 | |
| RICEGENE26571 | 487.4 | 832.8 | 2075.0 | 2515.4 | 182.6 | 399.0 | 643.0 | 818.4 | 11.0 | 18.8 | Similar to Small heat stress protein class CIII. [Os02t0782500-01] | Os02g0782500_AK119261 | |
| RICEGENE30579 | 44.2 | 258.5 | 134.4 | 16.6 | 8.9 | 10.5 | 16.8 | 14.0 | 126.1 | 234.1 | Similar to Cor14b protein precursor. [Os04t0610600-01] | Os04g0610600_AK073109 | |
| RICEGENE29807 | 198.5 | 466.9 | 1046.7 | 217.0 | 98.4 | 111.7 | 89.0 | 100.6 | 16.1 | 15.1 | Late embryogenesis abundant (LEA) group 1 family protein. [Os04t0589800-01] | Os04g0589800_AK063682 | |
| RICEGENE12551 | 22.1 | 179.9 | 43.8 | 21.1 | 11.3 | 15.1 | 17.7 | 33.9 | 8.0 | 11.3 | Late embryogenesis abundant (LEA) group 1 family protein. [Os06t0110200-01] | Os06g0110200_AK107973 | |
| RICEGENE25662 | 16.2 | 125.3 | 6.9 | 8.1 | 11.1 | 12.8 | 15.5 | 15.9 | 51.3 | | Late embryogenesis abundant (LEA) group 1 family protein. [Os06t0324400-01] | Os06g0324400_AK063726 | |
| RICEGENE23212 | 38.3 | 177.9 | 12.6 | 7.5 | 9.8 | 12.1 | 13.3 | 13.2 | 14.2 | | Late embryogenesis abundant (LEA) group 1 family protein. [Os08t0327700-01] | Os08g0327700_AK107930 | |
| RICEGENE16252 | 2974.7 | 4520.0 | 10863.8 | 1962.6 | 327.6 | 266.6 | 655.4 | 257.4 | 94.2 | 118.3 | Late embryogenesis abundant protein repeat containing protein. [Os01t0705200-01] | Os01g0705200_AK073837 | |
| RICEGENE20659 | 23.7 | 212.1 | 65.6 | 17.6 | 11.3 | 209.4 | 14.5 | 37.5 | | 140.9 | Late embryogenesis abundant protein repeat containing protein. [Os03t0168100-01] | Os03g0168100_AK121575 | |
| RICEGENE16505 | 197.3 | 117.4 | 176.7 | 30.6 | 31.0 | 17.8 | 67.4 | 17.0 | | 3.9 | Late embryogenesis abundant protein repeat containing protein. [Os02t0250600-01] | Os02g0250600_CI479812 | |
| RICEGENE11289 | 8068.1 | 6917.7 | 3970.1 | 323.3 | 696.9 | 479.7 | 3255.7 | 46.4 | | 261.4 | LEA-like protein. [Os05t0542500-02] | Os05g0542500_AK119713 | |
| Peroxidase | | | | | | | | | | | | | |
| RICEGENE05220 | 687.2 | 380.9 | 599.6 | 435.5 | 2291.9 | 1542.5 | 1325.2 | 1442.2 | 0.3 | 0.1 | Similar to Peroxidase BP 1 precursor. [Os01t0963000-04] | Os01g0963000_AK104277 | |
| RICEGENE02142 | 460.9 | 238.2 | 408.3 | 327.1 | 1507.3 | 987.2 | 860.3 | 956.3 | 0.4 | | Similar to Peroxidase BP 1 precursor. [Os01t0963000-01] | Os01g0963000_AK061131 | |
| RICEGENE11966 | 20.8 | 6.7 | 14.6 | 9.2 | 22.9 | 61.6 | 25.9 | 29.0 | 0.5 | 0.4 | Peroxidase (EC 1.11.1.7). [Os10t0109600- | Os10g0109600_AK061206 | |
| RICEGENE11639 | 348.1 | 48.2 | 81.3 | 47.5 | 298.8 | 30.1 | 182.8 | 56.1 | | 0.4 | Similar to Class III peroxidase 136. [Os12t0111800-00] | Os12g0111800_CI552210 | |

| | | | | | | | | | | | | |
|---|---|---|---|---|---|---|---|---|---|---|---|---|
| RICEGENE15706 | 168.8 | 378.5 | 12.5 | 320.4 | 393.0 | 2732.4 | 20.2 | 1300.3 | 0.5 | 0.3 | Similar to Class III peroxidase GvPx2b (Fragment). [Os02t0240300-01] | Os02g0240300_AK104645 |
| RICEGENE35278 | 7.0 | 326.3 | 8.2 | 227.1 | 344.9 | 2355.5 | 12.5 | 1139.4 | 0.4 | | Similar to Class III peroxidase GvPx2b (Fragment). [Os02t0240300-01] | Os02g0240300_AK067000 |
| RICEGENE08929 | 7.6 | 6.3 | 20.5 | 8.5 | 10.4 | 106.7 | 18.5 | 14.1 | 0.3 | 0.1 | Haem peroxidase family protein. [Os06t0521500-01] | Os06g0521500_AK061144 |
| RICEGENE17013 | 2295.9 | 2069.0 | 1372.2 | 834.4 | 4948.1 | 3751.8 | 3500.3 | 1745.8 | 0.3 | 0.0 | Similar to Class III peroxidase GvPx2b (Fragment). [Os06t0546500-01] | Os06g0546500_AK073833 |
| RICEGENE24991 | 16.2 | 17.5 | 17.2 | 9.3 | 77.9 | 14.7 | 14.8 | 21.8 | 0.5 | 0.5 | Haem peroxidase, plant/fungal/bacterial family protein. [Os07t0104500-01] | Os07g0104500_AK108824 |

Reference


1.The Plant Journal (1996) 9,217-227

2.BMC Genomics 2008, 9:550

3.Plant Molecular Biology 14: 29-39, 1989.

4.Cell Stress and Chaperones (2013) 18:427–437

5.The Plant Journal (2003) 33, 751-763

6.The ScientificWorld Journal Volume 2013, Article ID 397401, 9 pages

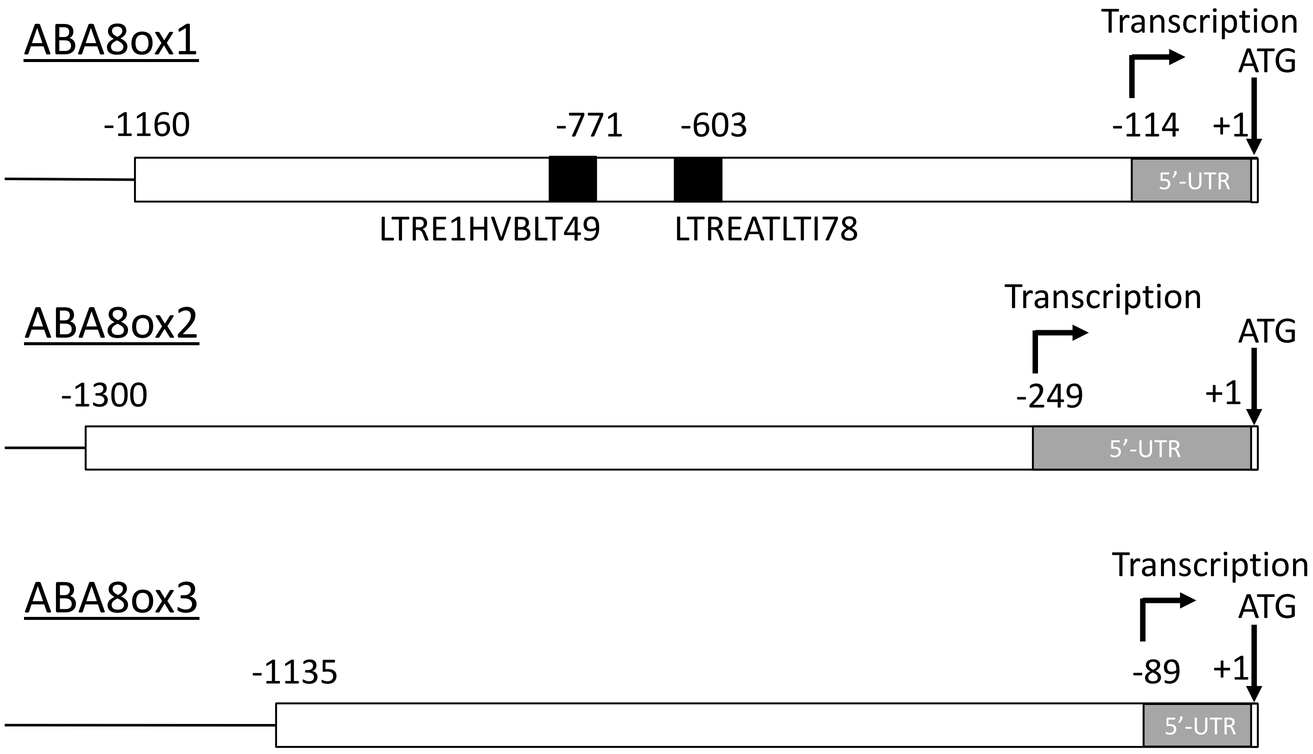


**Supplementary Figure S1.** Distribution of *cis*-acting elements in the promoter region of *OsABA8ox1, OsABA8ox2, and OsABA8ox3*. The black box indicates LTRE1HVLT49 and LTREATLTI78, described as low temperature responsive elements in the PLACE (http://www.dna.affrc.go.jp/PLACE/) database.

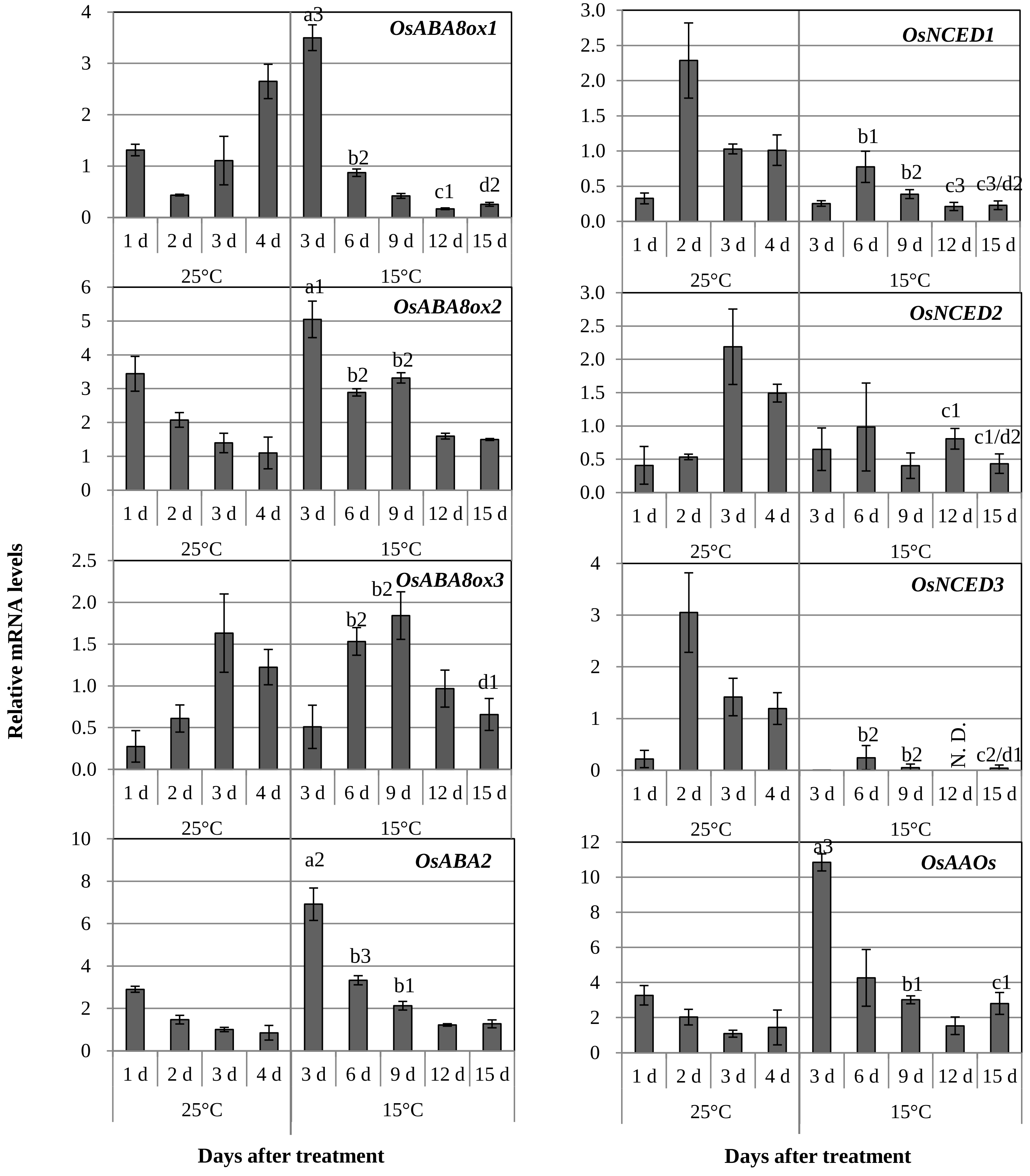


**Supplementary Figure S2.** Expression of *OsABA8oxs*, *OsNCEDs*, *OsABA2*, and *OsAAOs* under long-term cold stress. Rice seedlings after imbibition were transferred to and grown hydroponically at 15° C for 15 d. Values are means ± SD of three biological replicates. Significant differences between treated and control seedlings were determined by Student's *t*-tests. The letters a1, a2, and a3 represent $P < 0.05$, $P < 0.01$, and $P < 0.001$, respectively, between day 3 at 15° C and day 1 at 25° C. The letters b1, b2, and b3 represent $P < 0.05$, $P < 0.01$, and $P < 0.001$, respectively, between days 6 and 9 at 15° C and day 2 at 25° C. The letters c1, c2, and c3 represent $P < 0.05$, $P < 0.01$, and $P < 0.001$, respectively, between days 12 and 15 at 15° C and day 3 at 25° C group. The letters d1, d2, and d3 represent $P < 0.05$, $P < 0.01$, and $P < 0.001$, respectively, between day 15 at 15° C and day 4 at 25° C.

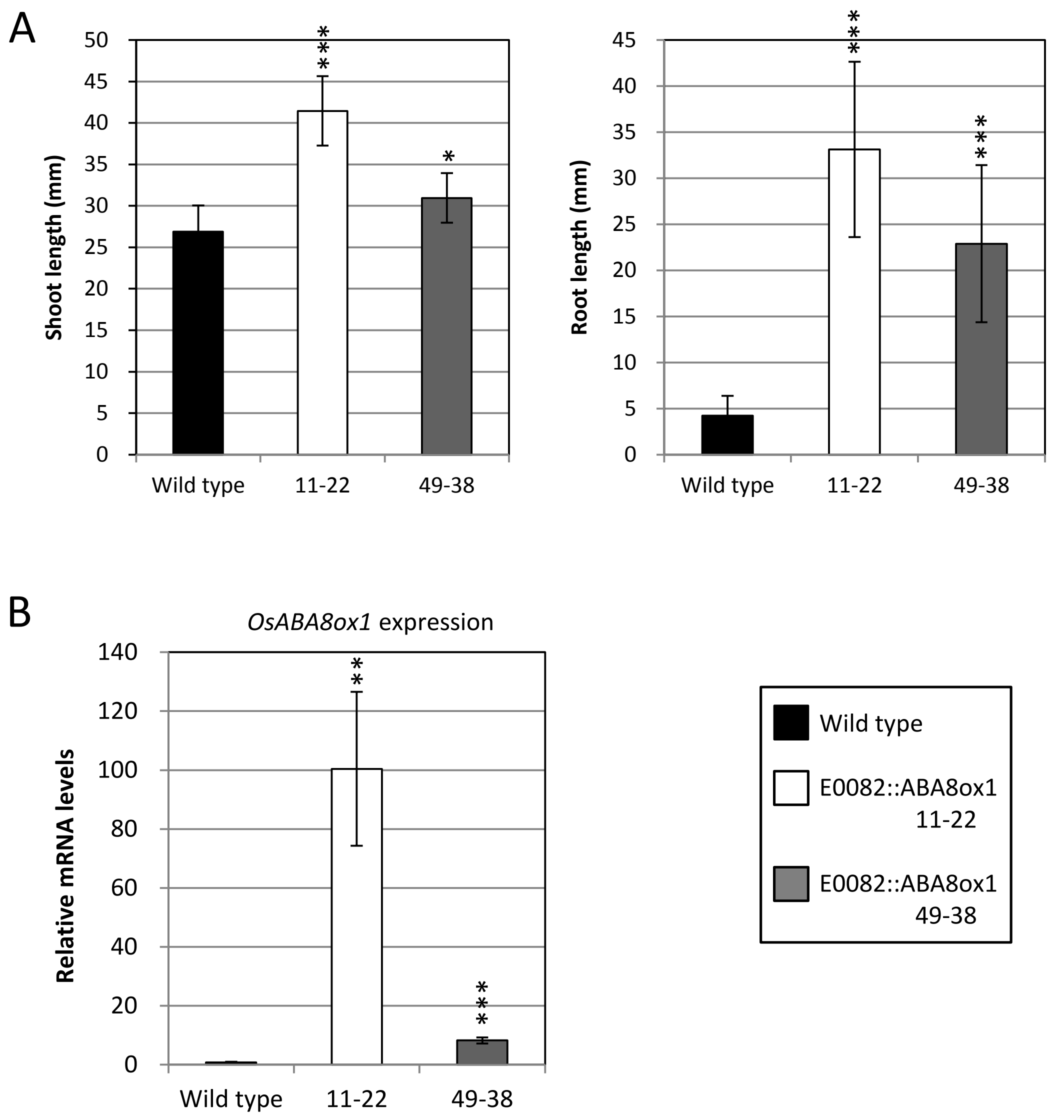


**Supplementary Figure S3.** Growth and *OsABA8ox1* gene expression of E0082::ABA8ox1 11-22 and E0082::ABA8ox1 49-38. Gene expression values are means ± SD of three biological replicates. Significant differences between transgenic and wild-type seedlings were determined by Student's *t*-tests. ** and *** represent $P < 0.01$ and $P < 0.001$, respectively.

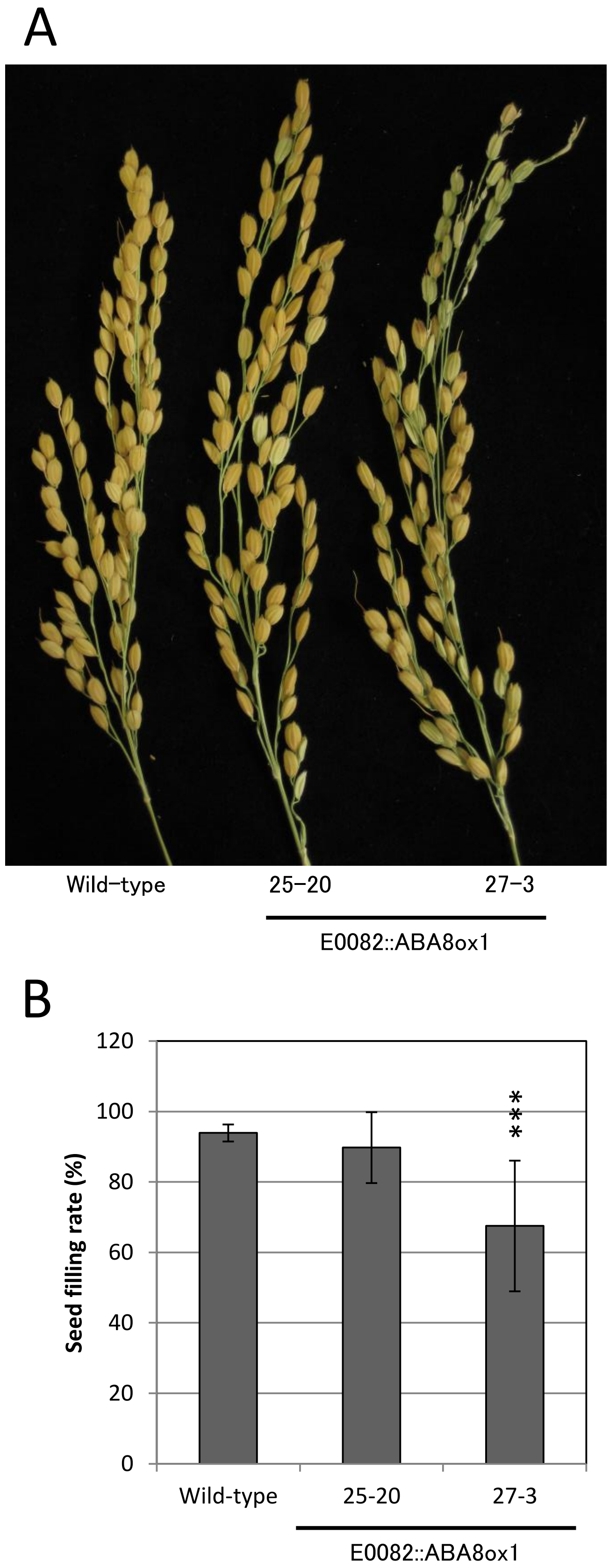


**Supplementary Figure S4.** Seed fertility rate of E0082::ABA8ox1 25-20 and E0082::ABA8ox1 27-3. Typical images (A) and seed fertility rate (B) of wild-type, E0082::ABA8ox1 25-20, and E0082::ABA8ox1 27-3. Twenty rice plants were grown in soil in a greenhouse for 4 months (14 h light, 25° C; 10 h dark, 20° C). Seeds were harvested from completely ripened plants. The number of filling and empty seeds was counted, and the filling seed rate was calculated. The experiments were repeated two times. Significant differences relative to the wild type were determined by Student's *t*-tests. ***, $P < 0.001$.
